# High‐speed bioimpedance‐based gating system for radiotherapy: Prototype and proof of principle

**DOI:** 10.1002/acm2.14491

**Published:** 2024-08-28

**Authors:** Mikhail A. Belikhin, Alexander P. Chernyaev, Alexander A. Pryanichnikov

**Affiliations:** ^1^ JSC Protom Protvino Russian Federation; ^2^ Lomonosov Moscow State University Moscow Russian Federation; ^3^ Division of Biomedical Physics in Radiation Oncology German Cancer Research Center (DKFZ) Heidelberg Germany

**Keywords:** respiratory gated radiotherapy, thoracic bioimpedance, tumor motion

## Abstract

**Purpose:**

To investigate a novel bioimpedance‐based respiratory gating system (BRGS) designed for external beam radiotherapy and to evaluate its technical characteristics in comparison with existing similar systems.

**Materials and methods:**

The BRGS was tested on three healthy volunteers in free breathing and breath‐hold patterns under laboratory conditions. Its parameters, including the time delay (TD) between the actual impedance change and the gating signal, temperature drift, root mean square (RMS) noise, and signal‐to‐noise ratio (SNR), were measured and analyzed.

**Results:**

The gate‐on TD and the gate‐off TD were found to be 9.0 ± 2.0 ms [mean ± standard deviation (M ± SD)] and 7.2 ± 1.3 ms, respectively. The temperature drift of the BRGS output signal was 0.02 Ω after 30 min of operation. RMS noise averaged 0.14 ± 0.05 Ω (M ± SD) for all subjects and varied from 0.08 to 0.20 Ω with repeated measurements. A significant difference in SNR (*p* < 0.001) was observed between subjects, ranging from 4 to 15.

**Conclusion:**

The evaluated bioimpedance‐based gating system showed a high performance in real‐time respiratory monitoring and may potentially be used as an external surrogate guidance for respiratory‐gated external beam radiotherapy. Direct comparison with commercially available systems, 4D correlation studies, and expansion of the patient sample are goals for future preclinical studies.

## 1 INTRODUCTION

In external beam radiotherapy, intrafractional respiration‐induced tumor motion is a challenge in the treatment of thoracic and abdominal cancers.^1^ This motion distorts the dose distribution, significantly increasing the dose to healthy tissue and decreasing dose homogeneity in the target volume. Active motion mitigation techniques, including breath‐hold (BH) and gating, require real‐time monitoring of the tumor motion,^2^ to synchronize the medical accelerator and the beam delivery with it.

Non‐invasive external surrogate systems such as optical surface tracking and spirometry are commonly used for real‐time monitoring. These systems track indirect parameters that correlate with tumor motion based on four‐dimensional (4D) imaging.^3^ Thoracic electrical impedance measurement^4^ is an alternative that may address some of the limitations of optical and spirometric systems. Bioimpedance changes may more accurately reflect tumor motion in the lung and liver^5^ and allow monitoring despite paradoxical chest wall or diaphragm motion.^6^ The direct attachment of bioimpedance systems to the patient's body makes them independent of patient positioning or treatment room elements,^7^ compatible with immobilization devices, e.g., a compression shell,^8^ and capable of monitoring respiratory and cardiac motion simultaneously,^9^ essential for double‐gating treatments.^10^

For respiratory‐gated radiotherapy, the signal‐to‐noise ratio (SNR)^11^ and time delay (TD)^12^ of the external surrogate system are critical. These factors affect the resolution of the system and the uncertainty of the gate signal generation. The AAPM TG‐142 report^15^ recommends a TD of less than 100 ms to maintain positional uncertainty within 2 mm for tumor velocities up to 20 mm/s. However, lung tumor motion can reach 100 mm/s,^14^ potentially causing significant positional uncertainty and requiring systems with minimal TD for optimal response and accuracy.

Kohli et al.^9^ and Koivumäki et al.^15^ have previously proposed two bioimpedance‐based monitoring systems for radiotherapy and positron emission tomography (PET). Kohli et al.^9^ described a prototype designed and tested for dual‐gated radiotherapy, while Koivumäki et al.^15^ presented an in‐house system for gated PET and emphasized its clinical application.

This study presents a novel bioimpedance respiratory gating system (BRGS) designed for radiotherapy that allows real‐time respiratory monitoring and gating signal generation. The prototype BRGS was technically evaluated for TD and temperature drift, and its SNR was evaluated in three healthy volunteers.

## 2 METHODS AND MATERIALS

### 2.1 Principle of operation

**FIGURE 1:**
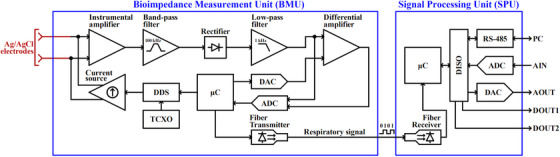
The block diagram of the bioimpedance respiratory gating system (BRGS) electronics.

**FIGURE 2:**
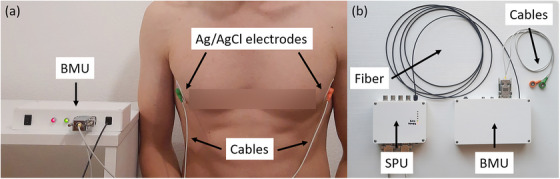
(a) Configuration of electrode placement during respiratory monitoring in a subject and (b) the prototype of the bioimpedance respiratory gating system (BRGS) consisting of a bioimpedance measurement unit (BMU) and a signal processing unit (SPU).

The BRGS consists of a bioimpedance measurement unit (BMU) and a signal processing unit (SPU) (Figure 1). The battery‐powered BMU measures thoracic impedance and is designed to ensure patient safety by isolating it from electromagnetic interference. It uses a current source to inject an alternating current with a root mean square (RMS) value of 850 µA and a frequency of 100 kHz into the patient's chest via two disposable self‐adhesive Ag/AgCl electrodes (Figure 2a). The system uses a direct digital synthesizer (DDS) controlled by a microcontroller (µC) via a serial peripheral interface (SPI) to adjust the amplitude and frequency of the current. The resulting potential difference is amplified, filtered, and rectified to produce a respiratory signal. This signal is then digitized by an analog‐to‐digital converter (ADC) at a rate of 1000 samples per second (S/s) and averaged over 5 ms by the µC. The processed signal is transmitted to the SPU at 200 S/s via a fiber optic line.

The SPU houses a µC that processes the respiratory signal to generate real‐time gating signals. It includes an amplitude gating algorithm, analog input/output (AIN/AOUT) for signal acquisition and generation, and digital outputs for gating signals (DOUT1/2). The SPU's components are electromagnetically isolated (DISO), and it interfaces with a PC via RS‐485. The BRGS software, developed in LabWindows/CVI (National Instruments, Austin, USA), configures gating parameters, and visualizes respiratory signals at 10 samples per second (S/s).

Calibration of the BRGS to convert the voltage signal to impedance was performed using an electrically equivalent tissue model (EETM)^16^ that simulates human tissue with a passive circuit. The EETM included parallel capacitive (0.1 µF) and resistive (22 kΩ) components with an adjustable serial resistive (1 kΩ) component, yielding a calibration factor of 4.33 ± 0.04 Ω/V [mean ± standard deviation (M ± SD)] with less than 1.6% uncertainty over a range of 100−900 Ω.

### 2.2 Time delay (TD)

**FIGURE 3:**
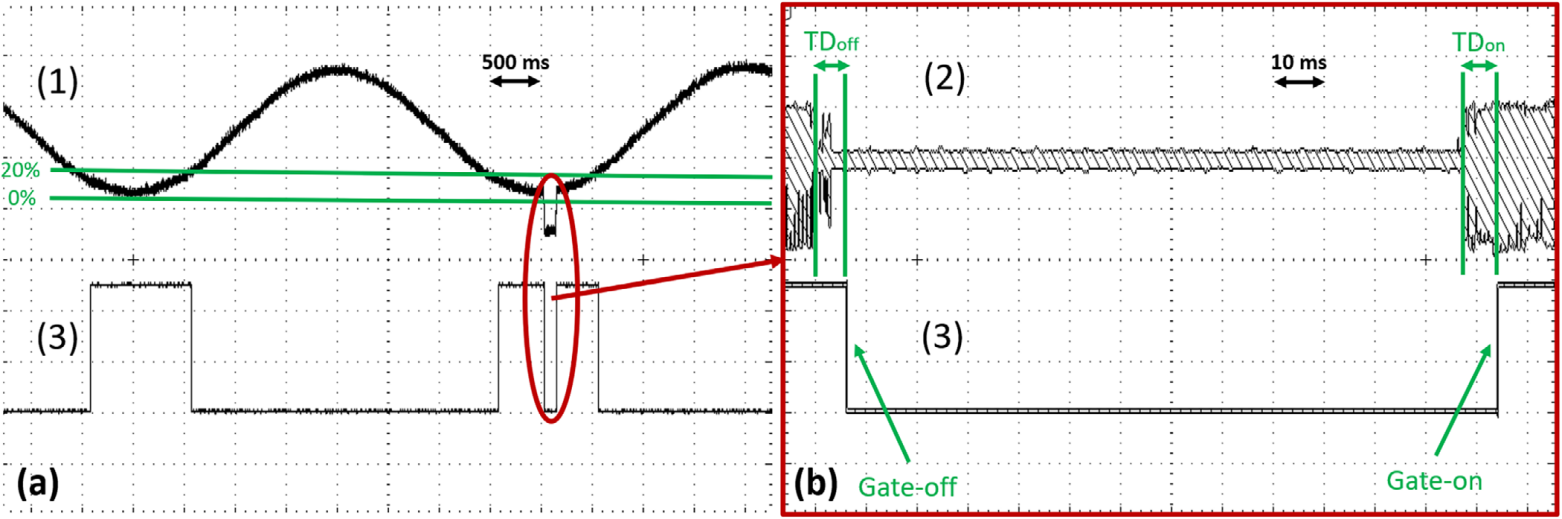
(a) The gating time diagram showing the respiratory signal (1), which was obtained at the differential amplifier output (see Figure 1) when the test signal (2) is applied to the BRGS input, and the gating signal (3) generated at DOUT1 of the SPU (see Figure 1). (b) The time diagram of the TD measurement showing the magnified area (red ellipse) of the signals where irregularities occurred, but instead of the respiratory signal (1) at the differential amplifier output, the test signal (2) applied to the instrumental amplifier input (see Figure 1) and recorded in Peak Detect mode is shown. The green horizontal lines show the gating window, and the vertical lines show the gate‐on TD (TD_on_) and the gate‐off TD (TD_off_). Both graphs are based on oscilloscope screenshots.

To evaluate the TD between the actual impedance change and the gating signal, the BMU current source was disabled and a test signal was applied directly to the BRGS. This signal was generated by an UTG1010A (Uni‐Trend Technology, Dongguan, China) and mimicked an impedance change. It had an amplitude of 100 mV and a frequency of 100 kHz with a sinusoidal modulation depth of 50% [Figure 3b, signal (2)]. The resulting sinusoidal respiratory signal had a period of 4 s [Figure 3a, signal (1)]. The BRGS operated in amplitude gating [Figure 3a, 3b, signal (3)] within a window of 0%−20% of the peak‐to‐peak (pk‐pk) amplitude of the respiratory signal.

The test and gating signals were acquired by a DS7072 (OWON Technology, Zhangzhou, China). At the time of gating signal generation, the UTG1010A was briefly turned off for 100−200 ms. This simulated an irregularity in the respiratory signal that caused the gating signal to be turned off. The gate‐on/off TD (TD_on_/TD_off_) was calculated by measuring the time difference between the rise/fall of the test signal and the rise/fall of the gating signal. Both TD_on_ and TD_off_ were measured 10 times to assess the statistical variance. In addition, one‐way analysis of variance (ANOVA) with a significance level of *p* < 0.05 was used to statistically compare the TD obtained at gate‐on and gate‐off.

### 2.3 Temperature drift

The temperature drift of the injected current, its frequency, and the respiratory signal (Figure 1, AOUT) were measured for 30 min after power‐on with the input electrodes short‐circuited at an ambient temperature of 25°C. A U3401A digital multimeter (Keysight, Santa Rosa, USA) was used to measure the injected current and the respiratory signal. Frequency was measured using the UTG1010A.

### 2.4 Signal data acquisition

The BRGS was tested on three subjects (healthy volunteers) in three scenarios (S) to measure respiratory and gating signals: (S1) BH for 10 s at the end of expiration; (S2) free breathing (FB) for 60 s with 0%−20% gating; (S3) FB for 50 s with voluntary BH for 10 s. The S1 was used to acquire the noise signal because the organs were almost immobile during BH, which allows minimizing the change in impedance due to respiratory motion. The S2 was used to acquire the respiratory and gating signals during FB. The respiratory signal together with the noise signal allowed to evaluate the SNR. The gating signal demonstrated the operation of the gating algorithm integrated in the SPU. The S3 was used to evaluate the BRGS for the breathing pattern implemented in the deep‐inspiration breath‐hold (DIBH) treatment. For each scenario, signal data acquisition was repeated three times to evaluate statistical variations in breathing patterns.

Respiratory monitoring was performed with the subjects in the upright position. Two new Ag/AgCl electrodes FS‐521 (Skintact, Innsbruck, Austria) were placed on the chest of each subject 5 min before the start of the measurement. The 5‐min delay was introduced to ensure robust contact between the electrode gel and the skin and to minimize contact impedance drift. The lateral configuration of the electrode placement was used, which provides the greatest sensitivity to the lung region.^17^ Electrode placement was similar for all subjects as shown in Figure 2a. For repeated measurements, the spent electrodes were removed and replaced with new electrodes in approximately the same position and the measurement was repeated after 5 min. The repeatability of the electrode position between different measurements was within ± 1 cm. The BRGS was also preheated for 15 min to reduce temperature‐related variations. No visual feedback was provided to the subjects during data collection.

### 2.5 Signal data analysis

**FIGURE 4:**
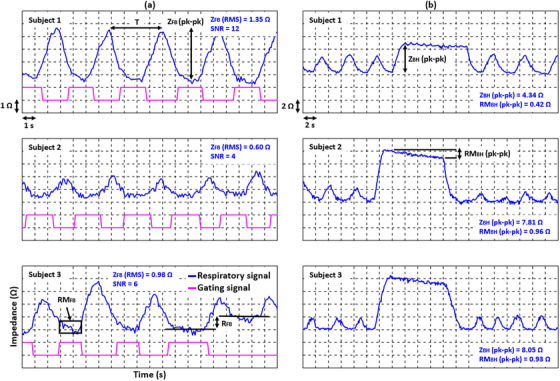
Examples of the respiratory and gating signals (a) for the free breathing (FB) pattern with gating and (b) for the breath‐hold (BH) pattern for three subjects. The RMS amplitude (Z_FB_) and SNR for the FB signals, and the peak‐to‐peak (pk‐pk) amplitude (Z_BH_) and pk‐pk intra‐BH residual motion (RM_BH_) for the BH signals are shown.

Thoracic impedance as a function of time was obtained from the noise, FB, and BH signal data sets. The analysis was performed using several metrics. For the noise signals, only the RMS amplitude (Z_N_) was evaluated. For the FB respiratory signals, the period (T), the RMS FB amplitude (Z_FB_), the pk‐pk FB amplitude, the RMS intra‐gate residual motion (RM_FB_), and the inter‐gate reproducibility (R_FB_) of the impedance between individual gating pulses were evaluated. For the BH signals, the pk‐pk BH amplitude of deep inspiration (Z_BH_) and the pk‐pk intra‐BH residual motion (RM_BH_) were evaluated. In addition, ANOVA was used to assess the significance of differences in the respiratory signal analysis metrics between subjects. All metrics are shown in Figure 4.

The period of the FB signal was estimated as the time difference between adjacent signal peaks in a single respiratory cycle. A pk‐pk amplitude was calculated as the difference between the maximum and minimum values of the respiratory signal for each single respiratory cycle (for the FB signal) or for a single deep inspiration (for the BH signal). The RMS values of the metrics, including Z_N_, Z_FB_, RM_FB_, and R_FB_, were calculated from the acquired data set according to Equation (1):

(1) $$Z_{\mathrm{RMS}}=\sqrt{\frac{\sum_{i=1}^{n}{\left(Z_{i}-Z_{m}\right)}^{2}}{n}}\;$$

where Z_i_—instantaneous signal value, Z_m_—mean signal value, n—number of instantaneous values used to calculate the mean. The SNR was calculated as the ratio of the RMS amplitude of the respiratory signal and the noise signal using Equation (2):

(2) $$\mathrm{SNR}\;=\frac{Z_{\mathrm{FB}}}{Z_{N}}\;$$

Metrics characterizing residual motion for the FB and BH signals, including RM_FB_ and RM_BH_, were evaluated as percentages relative to the RMS FB amplitude and the pk‐pk BH amplitude, respectively. To calculate inter‐gate reproducibility, the FB signal was averaged within the gating window, and reproducibility was defined as the RMS spread of these values between individual gating pulses (Equation 1).

## 3 RESULTS

The gate‐on TD and the gate‐off TD were found to be 9.0 ± 2.0 ms (M ± SD) and 7.2 ± 1.3 ms, respectively. The gate‐on TD was slightly longer than the gate‐off TD (*p* ≈ 0.02). The temperature drift of the BRGS output signal was 0.02 Ω after 30 min of operation. The temperature drift of the injected current and its frequency were 280 and 28 ppm, respectively. The characteristics of the subjects and the results of the analysis of the respiratory signals are summarized in Table 1.

**TABLE 1:** Summary of the respiratory signal analysis results. For T, Z_N_, Z_FB_, RM_FB_, Z_BH_, and RM_BH_, data are expressed as mean ± standard deviation (M ± SD), and for SNR—As M ± SD and range of values.

Subject	*T* (s)	Z_N_ (Ω) (RMS)	Z_FB_ (Ω) (RMS)	RM_FB_ (Ω) (RMS)	Z_BH_ (Ω) (pk‐pk)	RM_BH_ (pk‐pk)	SNR	
							M ± SD	Range
1. Male, 30 y.o., 175 cm, 65 kg	4.1 ± 0.4	0.11 ± 0.02	1.2 ± 0.1	0.29 ± 0.04	4.0 ± 0.3	0.4 ± 0.1	11 ± 2	9–15
2. Male, 55 y.o., 176 cm, 70 kg	3.9 ± 0.4	0.16 ± 0.01	0.7 ± 0.1	0.16 ± 0.03	7.1 ± 0.7	0.9 ± 0.2	5 ± 1	4–5
3. Female, 56 y.o., 159 cm, 61 kg	3.9 ± 0.6	0.16 ± 0.04	1.3 ± 0.4	0.22 ± 0.05	7.8 ± 0.3	0.7 ± 0.3	8 ± 3	5–13

RMS noise averaged 0.14 ± 0.05 Ω (M ± SD) for all subjects and varied from 0.08 to 0.20 Ω with repeated measurements. No significant difference in RMS noise was observed between subjects (*p* ≈ 0.06). The FB signals were clearly observed, and the gating signals were generated for all subjects (Figure 4a). The RMS FB amplitude varied significantly between subjects (*p*≈0.04), ranging from 0.70 Ω (subject 2) to 1.66 Ω (subject 3). The pk‐pk FB amplitude was found to be 3.6 ± 0.3 Ω (M ± SD), 2.1 ± 0.3 Ω, and 2.7 ± 0.6 Ω for subjects 1, 2, and 3, respectively. A significant difference in SNR was also observed between subjects (*p* < 0.001). Subject 1 had the highest SNR of up to 15, while subject 2 had the worst SNR of all subjects, down to 4. Intra‐gate residual motion was found to average 21 ± 4% (M ± SD) for all subjects. The inter‐gate reproducibility between individual gating pulses, obtained for 60 s of FB, was manifested as unsystematic variations in impedance. These variations averaged 0.16 ± 0.13 Ω (M ± SD) across subjects, with a maximum value of up to 1 Ω (Figure 4a, subject 3) in occasional cases (< 5% of all respiratory cycles included in the analysis).

The BH signals were also clearly observed in all subjects (Figure 4b). The pk‐pk BH amplitude varied between the subjects in the range of 3.73–8.05 Ω. A slight intra‐BH decrease in impedance was observed for all subjects in all repeated measurements. The pk‐pk intra‐BH residual motion was found to be of 11 ± 2% (M ± SD) for all subjects.

There was a statistically significant difference in respiratory periods between subjects (*p* ≈ 0.03), but the mean period for all subjects was 4.0 ± 0.1 s (M ± SD). FB of subject 3 appeared to be irregular compared to subjects 1 and 2, as evidenced by period and pk‐pk FB amplitude variations of up to 15% (SD) and 22% (SD), respectively. FB of subject 1 was the most regular, resulting in period and pk‐pk FB amplitude variations of 10% (SD) and 8% (SD), respectively.

## 4 DISCUSSION

The BRGS was stable in both FB and DIBH patterns in all subjects. Gating signals were generated in > 95% of respiratory cycles despite respiratory irregularities of up to 22%. Typical impedance changes were 2−4 and 4−8 ohms for FB and BH, in agreement with Koivumäki's study.^17^ Residual motion within the gating window averaged 21% of the motion amplitude, corresponding to the established 0%−20% window.

The TD introduced by the BRGS between the actual impedance change and the gating signal was 10 ms. It was less than the TD from Kohli's bioimpedance system^12^ (33 ms), the AZ‐733 V/733VI (Anzai Medical, Tokyo, Japan) (38 ms),^18^ and the various optical systems.^11^ The accelerator‐specific TD between actual motion and beam on/off should be measured, as it may vary significantly between facilities.^19^

The SNR was highly subject dependent and ranged from 4 to 15, but was higher than that of Kohli's system (≈4). The SNR may be the parameter specific to the electrode position, as the sensitivity of a bioimpedance device is a function of the configuration of the electrode placement.^17^ We studied the lateral configuration recommended for the highest sensitivity, but in the clinical workflow, it may be necessary to use a different electrode position to avoid interference of the electrode with the beam delivery, which may result in a decrease in SNR. Although the electrodes were placed in the same position within ± 1 cm for repeated measurements, the statistical variation in SNR was found to be 18% (subject 1) to 38% (subject 3). These variations may be related to the natural breathing irregularities and the spread of the RMS noise values (see Table 1) rather than to the repeatability of the electrode position. In addition, the noise may have been overestimated because systematic impedance changes associated with residual organ motion during BH and cardiac motion contributed to the noise calculation, whereas we defined the noise as random fluctuations. In the future, the repeatability of the BRGS can be quantified by simultaneous monitoring with an external optical or spirometry system equipped with visual feedback, allowing the subject to breathe in the same way each time, minimizing irregularities.

The current study focused on respiratory monitoring and performance measurement for the BRGS, so we used the conventional Ag/AgCl electrodes and metal connectors intended for electrocardiography measurements. Although these electrodes are widely used, their implementation in radiotherapy and imaging is limited due to several disadvantages, including significant CT artifacts^20^ and interference with beam delivery. When beams of >10 MeV photons and 70−250 MeV protons are used, secondary particles, particularly neutrons, can be produced in high‐Z components of the electrodes and connectors, resulting in additional non‐therapeutic dose. A Monte Carlo simulation can be performed with the geometry of the electrodes and cables to evaluate the dose both when the beam passes directly through the electrodes/cables and when scattered radiation strikes them.

To potentially minimize these effects, the BRGS utilized a two‐electrode measurement scheme, instead of the most commonly used four‐electrode scheme,^20^ to reduce the number of electrodes and connecting cables on the chest. The two‐electrode scheme provided an acceptable SNR of 10 on average, but the signal baseline drift was noticeable immediately after the new electrodes were placed on the chest, so we waited 5 min for the drift to stop before starting to record the respiratory signal. The four‐electrode scheme can potentially minimize this drift by increasing the absolute measurement accuracy by separating the contribution of contact impedance from the thoracic volume.^21^

For clinical implementation of the BRGS in radiotherapy, radiolucent carbon^20^ or conductive plastic^22^ electrodes and connectors are preferred because they allow minimization of CT artifacts^20^, ^22^ and do not significantly affect signal detection.^9^ Improvements to the BRGS in terms of compatibility with low‐Z electrodes and the four‐electrode scheme should be made before clinical implementation. A two‐electrode scheme with the conventional Ag/AgCl electrodes may also be a potential solution when radiolucent electrodes are not available, but its use in a clinical setting may be difficult.

The BRGS can be used for both DIBH and respiratory‐gated treatment. The implementation of battery power and optical isolation, as well as the integration of the gating algorithm into the microcontroller code, allowed high performance and good SNR to be achieved. Compared to other bioimpedance systems,^9^, ^15^ the BRGS not only provides respiratory monitoring, but also generates the gating signal for the accelerator and has a real‐time user interface. The BRGS appears to be more suitable for fixed beam treatment facilities, such as the P‐Cure proton therapy facility (P‐Cure, Shilat, Israel), where the patient can move over a large area from the nozzle to the CT scanner.

The BRGS also has several disadvantages and limitations. Validation of the BRGS requires a special 4D phantom made of electrically conductive materials, which does not allow it to be used with commercially available 4D phantoms. The influence of a pacemaker on the operation of bioimpedance gating systems has not been sufficiently investigated. The cardiac signal is not separated from the respiratory signal in the tested BRGS prototype. Finally, the BRGS provides only respiratory monitoring, whereas some optical systems allow surface‐based alignment of the patient's position.

## 5 CONCLUSION

The evaluated bioimpedance‐based gating system showed a high performance in real‐time respiratory monitoring and may potentially be used as an external surrogate guidance for respiratory‐gated external beam radiotherapy. However, prior to clinical implementation, it is necessary to investigate the correlation of tumor motion with changes in thoracic bioimpedance, develop a bioimpedance‐specific phantom, perform a direct comparison of the bioimpedance system with commercially available systems used in clinical practice, and increase the population sample on which to test it.

## AUTHOR CONTRIBUTIONS

Mikhail A. Belikhin participated in the preparation of equipment and measurements. Mikhail A. Belikhin, Alexander P. Chernyaev, and Alexander A. Pryanichnikov participated in data analysis and interpretation of results. Mikhail A. Belikhin and Alexander A. Pryanichnikov drafted the original manuscript. All authors critically revised the manuscript and approved the submitted version.

## CONFLICT OF INTEREST STATEMENT

The authors have no conflict of interest to disclose.

## ETHICS STATEMENT

Ethics approval and consent for participation: approval from the Ethics Committee of the Experimental Medicine Research Institute for Complex Issues of Cardiovascular Diseases (Ф 2.2./01‐06). All subjects signed an informed consent form.
